# Determination of suitable housekeeping genes for normalisation of quantitative real time PCR analysis of cells infected with human immunodeficiency virus and herpes viruses

**DOI:** 10.1186/1743-422X-4-130

**Published:** 2007-12-03

**Authors:** Sarah Watson, Sarah Mercier, Chris Bye, John Wilkinson, Anthony L Cunningham, Andrew N Harman

**Affiliations:** 1 Centre for Virus Research, Westmead Millennium Institute, Sydney, Australia; 2 The Howard Florey Institute, University of Melbourne, Melbourne, Australia; 3 Biotron Limited, Centre for Immunology, St. Vincent's Hospital, Sydney, Australia

## Abstract

The choice of an appropriate housekeeping gene for normalisation purposes has now become an essential requirement when designing QPCR experiments. This is of particular importance when using QPCR to measure viral and cellular gene transcription levels in the context of viral infections as viruses can significantly interfere with host cell pathways, the components of which traditional housekeeping genes often encode. In this study we have determined the reliability of 10 housekeeping genes in context of four heavily studied viral infections; human immunodeficiency virus type 1, herpes simplex virus type 1, cytomegalovirus and varicella zoster virus infections using a variety of cell types and virus strains. This provides researchers of these viruses with a shortlist of potential housekeeping genes to use as normalisers for QPCR experiments.

## Background

Quantitative real-time PCR (QPCR) is an invaluable tool for the measurement of target nucleic acid sequences in clinical diagnostics and research. The technique is capable of the relative or absolute quantification of RNA or DNA sequences in a single sample over a large dynamic range with extreme sensitivity and accuracy. In virology and immunology QPCR is used for the relative measurement of virus and host transcription (gene expression) profiles in response to viral infection and in the quantification of viral load in clinical samples and in research [1].

The sensitivity and accuracy of results obtained by QPCR are dependent on a reliable reference within each sample to normalise for sample to sample and run to run variation [2]. These variations arise from differences in nucleic acid integrity, the efficiency of the reverse transcription of RNA to cDNA and the amount of sample loaded. References for normalising sample and run variation include the use of total nucleic acid concentrations, rRNA concentrations or the simultaneously measurement of the expression of an individual or select group of genes termed 'housekeeping' genes, which has become by far the most commonly used method and is the most reliable [3]. Housekeeping genes are used under the assumption that their expression is unchanged in response to the experimental conditions being investigated. Therefore a major factor when using QPCR is the selection of appropriate control genes, as any variation in the expression of the reference gene between sample groups will reduce the sensitivity of the assay to detect changes in the expression of genes of interest and may also produce artificial changes [3]. Traditionally glyceraldehyde 3-phosphate dehydrogenase (GAPDH) and β-actin (BACT) have been most commonly used, however many recent reports have shown that the expression of these genes can be variable under several experimental conditions, making them inappropriate for use as normalisers [4-7]. In reality no cellular gene maintains constant expression levels under all conditions and the evaluation of an appropriate control gene to normalise QPCR data is therefore an essential requirement when designing QPCR experiments using new experimental conditions[8]. This is especially the case when investigating the effects of viruses on host cell gene expression as viruses can interfere with host cell pathways, the components of which traditional housekeeping genes often encode. These include cell cycle, metabolism, DNA replication and transcription, in order to aid their replication cycle [9-11]. Studies have been carried out to determine reliable housekeeping genes in cells infected with a variety of viruses [7,12], however to date no such study has been carried out in cells infected with human immunodeficiency virus (HIV) or the herpes viruses, herpes simplex virus (HSV) and varicella zoster virus (VZV). Thus, in this study we have investigated the suitability of 10 commonly used housekeeping genes for use as QPCR normalisers in a variety of cell types infected with a variety of strains of HIV-1, HSV-1, VZV and cytomegalovirus (CMV) compared to mock infected cells.

## Results

A range of cell lines and primary cells were infected with HIV-1 (BaL, NL4.3, RFW strains), HSV-1 (NC-1 strain), CMV (Toledo and Towne strains) and VZV (Schenke strain) and harvested at the earliest time of maximal productive infection as illustrated in Table 1. The expression levels of 10 commonly used QPCR normalisation genes in both and mock and virally infected cells was determined by QPCR. The housekeeping genes investigated were beta-2microglobulin (B2M), peptidylprolyl isomerase A (PPIA), eukaryotic translation elongation factor 1 gamma (EEF1G), succinate dehydrogenase complex subunit A (SDHA), GAPDH, hydroxymethyl-bilane synthase (HMBS), TATA box-binding protein (TBP), 18s Ribosomal RNA (18sRNA), phosphoglycerate kinase 1 (PGK1), and BACT.

**Table 1:** Viral strains, infection and cell culture conditions

**Virus**	**Strain**	**Cell Type**	**Time Point**	**MOI**	**% Infection**
HIV-1	BaL	MDDC	48 hr	3	>30
HIV-1	NL4.3	HELA	48 hr	3	>70
HIV-1	NL4.3	HUT78	48 hr	3	>70
HIV-1	RFW	HUT78	48 hr	3	>80
HSV-1	NC1	HELA	18 hr	3	>95
HSV-1	NC1	HEP2	18 hr	3	>95
VZV	Schenke	MDDC	72 hr	0.3	>30
VZV	Schenke	HFF	72 hr	0.3	>60
CMV	Toledo	HFF	72 hr	3	>90
CMV	Towne	HFF	72 hr	3	>90

In order to accurately ascertain which housekeeping genes would be most reliable for use as normalisers in QPCR we subjected the data to analysis using the GeNorm tool[13] (Table 2). Using the results from the GeNorm analysis it was possible to list the housekeeping genes in order of reliability in the context of each viral infection (Table 3). 18sRNA was the least reliable reference gene as it had had the highest sum_v _value (13.26) which represents the standard deviation (SD) of reference gene expression over all viral infections investigated. Correspondingly it was either the least or second least reliable gene in 9 of the 10 experimental settings. Additionally BACT and HMBS also had high sum_v _values of 10.51 and 9.65 respectively, indicating that these genes are generally less reliable for use as normalisers for QPCR assays. According to this analysis the most reliable overall reference gene was PP1A with a sum_v _value of 7.41 and the most reliable gene in 7 of the 10 viral infections. Other genes with low sum_v _values were SDHA (8.91), GAPDH (8.74), TBP (9.06) and EEF1G (9.16), though in the context of HSV-1_NC1 _infected HELA cells EEF1G was identified as the least reliable gene. In the case of CMV an almost identical pattern in reliability was observed in infected primary HFF cells regardless of differences in the virus strain used and a very similar pattern was also seen in VZV_Schenke _infected cells despite differences in the origin of the primary cell types used for infection. Though the expression pattern of reference genes in HELA and HEP2 cells infected with HSV-1_NC-1 _were also similar, it is of note that in HEP2 cells GAPDH was the second most reliable gene whereas in HELA cells it was the third most unreliable. The expression pattern of reference genes in cells infected with HIV-1 was also similar though greater differences were detected compared to the other viruses, probably reflecting variability in both virus strain and cell type. Notable differences included 18sRNA being a relatively reliable gene in MDDCs infected with HIV_BaL_, but unreliable in other HIV infections and B2M being an unreliable reference gene in HIV-1_RFW _infected HUT78 cells only.

**Table 2:** Results of GeNorm analysis

Virus	Cell	**B2M**	**PP1A**	**EEF1G**	**SDHA**	**GAPDH**	**HMBS**	**18sRNA**	**PGK1**	**B-ACT**	**TBP**	**sum_RGC_**
HIV-1_BaL_	MDDC	0.98	0.78	1.04	1.26	0.83	0.84	0.98	1.09	1.45	0.97	***10.22***
HIV-1_NL4.3_	HELA	0.84	0.53	0.75	0.88	0.60	0.84	0.93	0.78	0.90	0.72	***7.77***
HIV-1_NL4.3_	HUT78	1.11	1.18	1.34	0.96	1.35	1.13	2.20	1.20	1.38	1.05	***12.90***
HIV-1_RFW_	HUT78	1.21	0.64	0.83	0.90	0.99	1.17	1.53	0.87	0.97	1.16	***10.27***
HSV-1_NC1_	HELA	0.80	0.65	1.33	0.62	0.91	0.59	0.89	0.60	0.92	0.74	***8.05***
HSV-1_NC1_	HEP2	0.41	0.42	0.44	0.50	0.37	0.38	0.60	0.41	0.57	0.54	***4.64***
VZV_Schenke_	MDDC	0.99	0.93	0.96	1.09	0.93	1.23	1.83	1.30	1.02	0.94	***12.22***
VZV_Schenke_	HFF	1.56	1.09	1.12	1.34	1.45	1.75	1.94	1.87	1.70	1.54	***15.36***
CMV_Towne_	HFF	0.65	0.58	0.60	0.60	0.82	0.60	1.14	0.58	0.74	0.63	***6.8***
CMV_Toledo_	HFF	0.86	0.61	0.75	0.76	0.71	0.90	1.22	0.73	0.86	0.77	***7.87***
	***sum*_*v*_**	***9.41***	***7.41***	***9.16***	***8.91***	***8.74***	***9.65***	***13.26***	***9.43***	***10.51***	***9.06***	

The standard deviations of reference gene expression as determined by GeNorm are shown. Abbreviations: Sum_v_: Sum of viral infection GeNorm values; sum_RGC_: sum of reference gene GeNorm values

**Table 3:** Reliability of references genes for each viral – cell pair

**Virus**	**HIV-1_BaL_**	**HIV-1_NL4.3_**	**HIV-1_NL4.3_**	**HIV-1_RFW_**	**HSV-1_NC1_**	**HSV-1_NC1_**	**VZV_Schenke_**	**VZV_Schenke_**	**CMV_Towne_**	**CMV_Toledo_**	**Overall**
**Cell**	**MDDC**	**HELA**	**HUT78**	**HUT78**	**HELA**	**HEP2**	**MDDC**	**HFF**	**HFF**	**HFF**	**-**

**1st**	PP1A	PP1A	SDHA	PP1A	HMBS	HMBS	PP1A*	PP1A	PP1A*	PP1A	**PP1A**
**2nd**	GAPDH	GAPDH	TBP	EEFIG	PGK1	GAPDH	GAPDH*	EEFIG	PGK1*	GAPDH	**GAPDH**
**3rd**	HMBS	TBP	B2M	PGK1	SDHA	PGK*	TBP	SDHA	GAPDH**	PGK1	**SDHA**
**4th**	TBP	EEFIG	HMBS	SDHA	PP1A	B2M*	EEF1G	GAPDH	EEF1G**	EEFIG	**TBP**
**5th**	B2M	PGK1	PP1A	BACT	TBP	PPIA	B2M	TBP	SDHA**	SDHA	**EEF1G**
**6th**	18sRNA	HMBS*	PGK1	GAPDH	B2M	EEF1G	BACT	B2M	TBP	TBP	**B2M**
**7th**	EEF1G	B2M*	EEFIG	TBP	18sRNA	SDHA	SDHA	BACT	B2M	B2M*	**PGK1**
**8th**	PGK1	SDHA	GAPDH	HMBS	GAPDH	TBP	HMBS	HMBS	BACT	BACT*	**HMBS**
**9th**	SDHA	BACT	18sRNA	B2M	BACT	BACT	PGK1	PGK1	HMBS	HMBS	**BACT**
**10th**	BACT	18sRNA	BACT	18sRNA	EEFIG	18sRNA	18sRNA	18sRNA	18sRNA	18sRNA	**18sRNA**

Where two or more genes were equally reliable these are labelled with * or **.

## Discussion

There are now many reports describing the unreliability of conventionally used housekeeping genes for the normalisation of QPCR data in certain experimental settings [4-7,14,15]. Careful consideration must therefore be carried out when choosing appropriate reference genes in QPCR experiments and the reliability of a panel of potential genes should be determined for individual experimental conditions[3,8]. Ideally the best two or three of these genes would then be used for normalisation purposes [16]. This is of particular concern to molecular virologists as most viruses modulate key cellular processes which may involve changing the expression of QPCR reference genes. Different viruses manipulate different cellular transcription pathways and the extent to which these pathways are affected will be dependent on the specific strain of virus and the cell type infected. Thus it is not possible to identify a single housekeeping gene for use in QPCR studies of viral infections. Nevertheless it is of benefit for researchers to be able to determine a shortlist of potential candidates. To date the variability of housekeeping gene expression has been studied in cells infected with SARS corona virus, yellow fever virus, human herpes virus-6, camelpox virus, CMV [7] and Epstein-Barr virus (EBV) [12]. However such a study has never been carried out using the key human pathogens HIV, HSV and VZV. Therefore in this study we have extended the published investigations by determining the suitability of 10 commonly used reference genes in the context of infection with various strains of HIV-1, HSV-1, CMV and VZV in a range of both primary and cultured cell lines using the GeNorm tool[13].

We found that overall 18sRNA was the least reliable gene studied and that BACT was also consistently unreliable. This correlates with the data from Radonic *et al *[7] who found BACT to be the most unreliable of 10 reference genes studied in a range of 5 viral infections. 18sRNA was not included in their study however. In contrast Bernasconi *et al *[12] found BACT had the lowest coefficient of variation of 451 housekeeping genes spotted on microarrays in EBV infection in B cells from patients with Burkitt's lymphoma. It is of note however that the γ-herpes virus EBV was not included in our study or of that of Radonic *et al *though the β-herpes virus CMV and α-herpes viruses HSV-1 and VZV were. In agreement with Radonic *et al *we found that PP1A was the most reliable reference gene across all infections. However in contrast to their finding that TBP was equally reliable as PP1A we found it to be the 4^th ^most reliable gene. Other reliable genes identified by our study included GAPDH and SDHA.

A comprehensive literature review of expression studies published in high-impact journals found that GAPDH, BACT, 18sRNA and 28sRNA were used as a single control gene in >90% of cases[13]. However, we found that two of these (18sRNA and BACT) were the least reliable in the context of the viral infections that we investigated. In contrast to all other infections, 18sRNA was not an unreliable control gene when MDDCs were infected with HIV-1_BaL. _In addition it is of note that although GAPDH was one of the most reliable reference genes identified overall, it was the third most unreliable gene in HELA cells infected with HSV-1_NC1_(whereas it was the second most reliable reference gene in HEP2 cells infected with the same strain of HSV-1). These findings therefore highlight the importance of re-evaluating the choice housekeeping genes when making even slight changes to experimental settings.

## Conclusion

In summary, PPIA, GAPDH and SDHA were the best QPCR control genes and we would recommend molecular virologists begin by short listing these genes when designing QPCR experiments. 18sRNA and BACT were consistently unreliable and should be used with caution in studies involving virally infected cells.

## Materials and methods

### Virus culture

HSV-1 strain NC1, CMV strains Towne and Toledo, VZV strain Schenke and HIV-1 strains BaL, NL4.3 and RFW were propagated according to standard protocols [16-20]. The cell types infected, MOI, time of infection and percentage of cells infected are shown in table 1. Cells were infected with each virus strain at a MOI and duration in order to achieve the maximal percentage of infected cells as determined by QPCR for all HIV-1, HSV-1 and CMV infected cells, [21] and by flow cytometry for VZV infected cells[22].

### RNA extraction and DNase treatment

Total RNA from cells derived from four independent experiments was extracted using the RNAqueous-Midi kit (Ambion, TA), quantified by UV spectroscopy and the integrity confirmed using an agilent 2100 bioanalyzer. 1 μg of the total RNA was then DNase treated using 1 U RNase free DNase (Promega, Madison WI).

### cDNA synthesis

cDNA was produced using the Superscript III RT-PCR System (Invitrogen, Rockville, MD) according to the manufacturer's recommendations for oligo(dT)20 primed cDNA-synthesis. cDNA synthesis from RNA was performed using 1 μg of RNA, at 50°C. The cDNA was then treated with RNase H (Invitrogen) and diluted 1:100 before use for QPCR.

### Quantitative PCR

In order to measure housekeeping gene expression, cDNA was subject to QPCR using the platinum QPCR super mix kit (Invitrogen) and pre-designed certified LUX primers (Invitrogen) designed to amplify the following transcripts: B2M, PPIA, EEF1G, SDHA, GAPDH, HMBS, TBP, 18sRNA, PGK1, BACT. Fluorescent PCR amplicons were detected using a Stratagene Mx3005 QPCR thermocycler using 96-well microtiter plates in a final volume of 25 μl under the following cycle conditions: 50°C for 2 minutes, 95°C for 2 minute, 45 cycles of 95°C for 15 seconds 55°C for 30 seconds and 72°C for 30 seconds.

### Data analysis

The ΔΔCT [23] method was used for initial data analysis. Four biological replicates were used for each infection and a two tailed test with a significance level of 5% was used to measure the significance between sample replicates. Repeated measures analysis of variance was then used to test for the presence of interaction between the effects of the within experiment factors for housekeeping gene and viral status of the cell sample. Data analysis was also performed using the GeNorm tool[13].

## Abbreviations

QPCR, quantitative PCR; CMV, cytomegalovirus; HSV, herpes simplex virus; VZV, varicella zoster virus; B2M, beta-2microglobulin; PP1A, peptidylprolyl isomerase A; EEF1G, eukaryotic translation elongation factor 1 gamma; SDHA, succinate dehydrogenase complex subunit A; GAPDH, glyceraldehyde 3-phosphate dehydrogenase; BACT, beta actin; HMBS, hydroxymethyl-bilane synthase; TBP, TATA box-binding protein; 18sRNA, 18s Ribosomal RNA; PGK1, phosphoglycerate kinase 1.

## Competing interests

The author(s) declare that they have no competing interests.

## Authors' contributions

AH, JW, CB and AC conceived of the study. AH designed and supervised the experiments, prepared the HIV infected MDDC extracts, and prepared the manuscript with CB. SW carried out the QPCR experiments and conducted data analysis with the help of SM. AC provided intellectual input, and grant funding. All authors read and approved the final manuscript.
